# Mapping of the DLQI scores to EQ-5D utility values using ordinal logistic regression

**DOI:** 10.1007/s11136-017-1607-4

**Published:** 2017-06-10

**Authors:** Faraz Mahmood Ali, Richard Kay, Andrew Y. Finlay, Vincent Piguet, Joerg Kupfer, Florence Dalgard, M. Sam Salek

**Affiliations:** 1 Department of Dermatology and Academic Wound Healing, Division of Infection and Immunity, School of Medicine, Cardiff University, Cardiff, CF14 4XN UK; 2 School of Pharmacy and Pharmaceutical Sciences, Cardiff University, Cardiff, UK; 3 Institute of Medical Psychology, Justus Liebig University, Giessen, Germany; 4 Department of Dermatology and Venereology, Skåne University Hospital, Lund University, 20502 Malmö, Sweden; 5 School of Life & Medical Sciences, Department of Pharmacy, University of Hertfordshire, Hatfield, UK; 6 Institute for Medicines Development, Cardiff, UK

**Keywords:** DLQI, Mapping, Utility values, Quality of life, EQ-5D, Ordinal logistic regression

## Abstract

**Purpose:**

The Dermatology Life Quality Index (DLQI) and the European Quality of Life-5 Dimension (EQ-5D) are separate measures that may be used to gather health-related quality of life (HRQoL) information from patients. The EQ-5D is a generic measure from which health utility estimates can be derived, whereas the DLQI is a specialty-specific measure to assess HRQoL. To reduce the burden of multiple measures being administered and to enable a more disease-specific calculation of health utility estimates, we explored an established mathematical technique known as ordinal logistic regression (OLR) to develop an appropriate model to map DLQI data to EQ-5D-based health utility estimates.

**Methods:**

Retrospective data from 4010 patients were randomly divided five times into two groups for the derivation and testing of the mapping model. Split-half cross-validation was utilized resulting in a total of ten ordinal logistic regression models for each of the five EQ-5D dimensions against age, sex, and all ten items of the DLQI. Using Monte Carlo simulation, predicted health utility estimates were derived and compared against those observed. This method was repeated for both OLR and a previously tested mapping methodology based on linear regression.

**Results:**

The model was shown to be highly predictive and its repeated fitting demonstrated a stable model using OLR as well as linear regression. The mean differences between OLR-predicted health utility estimates and observed health utility estimates ranged from 0.0024 to 0.0239 across the ten modeling exercises, with an average overall difference of 0.0120 (a 1.6% underestimate, not of clinical importance).

**Conclusions:**

This modeling framework developed in this study will enable researchers to calculate EQ-5D health utility estimates from a specialty-specific study population, reducing patient and economic burden.

**Electronic supplementary material:**

The online version of this article (doi:10.1007/s11136-017-1607-4) contains supplementary material, which is available to authorized users.

## Introduction

‘Health-related quality of life’ (HRQoL) data can be used to derive ‘Quality-Adjusted Life Years’ (QALYs), which are implemented in economic analyses to aid healthcare decision makers. The Dermatology Life Quality Index (DLQI) [1] is the most commonly used dermatology-specific HRQoL instrument [2]. In contrast, the European Quality of Life-5 Dimension (EQ-5D) [3] is a generic utility measure for use across all diseases [4] that provides health utility estimates, for comparison of disease burden, that has been little used in dermatology [5]. Both measures may be used together, though this may be burdensome, and integrating data from multiple measures presents challenges [6]: it is not clear whether two types of measures should inform the same decision [7].

There are several ‘mapping techniques’ [8] involving algorithms to predict health utility estimates from disease-specific measures. A linear model [9] was used to predict health utility estimates from the DLQI [10–12]. However, the methodology had limitations including small sample sizes and psoriasis-only populations, which may not be reliably used across a general dermatology population. Subsequent mapping models were derived using multiple linear regression [13] and bivariate/multivariate analysis [14], though the authors did not conduct formal validation to predict utility values and only went as far as predicting EQ-5D VAS or total scores. Blome et al. [14] pessimistically postulated that ‘any prediction of utilities with the DLQI and other variables regularly assessed in psoriasis studies will be vague and not of clinical relevance.’ However, Gray et al. [15] succeeded in mapping the Short-Form 12 to categorical EQ-5D responses using ordinal logistic regression (OLR).

There is a wealth of DLQI data from clinical studies over the last two decades without health utility estimate outputs recorded. Therefore, deriving this information from a dermatology-specific population would allow researchers to compare more disease-specific economic data across all conditions. The aim of this study was to create a mapping model using OLR to predict EQ-5D health utility estimates from DLQI scores, and we hypothesized that this can be done reliably. Previous unsatisfactory or failed attempts have used total DLQI scores to calculate health utility estimates for a cohort of patients, whereas a key aspect of OLR methodology is the use of data from individual DLQI items mapped to individual EQ-5D domains. We also aimed to produce health utility estimates utilizing the previous linear regression method employed by Currie and Conway [9] on our dataset, which is larger and more diverse, to compare the accuracy of the two distinct mapping techniques.

## Materials and methods

### The Dermatology Life Quality Index (DLQI)

The DLQI consists of ten items, with four possible responses to each item: “Very much,” “A lot,” “A little,” and “Not at all.” If any item for the DLQI was left unanswered, it was scored zero, following the developers’ instructions [16] (see Appendix). The two parts of item 7 were combined as a single item containing scores for both parts, as routinely done in calculating total scores. This allowed a uniform four-level ordinal response system for all DLQI items.

### The European Quality of Life-5 Dimension

The EQ-5D consists of two parts: a descriptive system and a visual analogue scale (VAS). The descriptive system contains five dimensions: “mobility,” “self-care,” “usual activities,” “pain/discomfort,” and “anxiety/depression.” The 3-level version EQ-5D-3L was used, for which there are three possible responses: “no problems,” “some problems,” and “extreme problems.” In our analysis, these outcomes were scored 1, 2, and 3.

### The data

Data from 4010 patients with skin diseases [17] were used. The patient dataset was accessed from an international multicenter observational cross-sectional study examining the association between depressive symptoms and dermatological conditions ranging from benign and malignant skin lesions to chronic inflammatory diseases such as psoriasis and lupus erythematosus [17]. The dataset (*n* = 4010) was filtered to exclude subjects with missing age, sex, DLQI, and EQ-5D data (11.7% in total). This resulted in a total of 3542 subjects. The socio-demographic characteristics for the entire patient dataset are given by Dalgard et al. [17], and have been summarized in Table 1. These patients were referred to outpatient dermatology clinics at various centers across Europe between 2011 and 2013. The full methodology has been previously described [17]. Each participant was examined and the main diagnosis recorded. Patients completed several questionnaires, including the DLQI and EQ-5D. This mapping study did not require additional ethics approval.

**Table 1:** Socio-demographic data for the complete dataset

	No. of patients
Country	
Belgium	222
Denmark	247
France	116
Germany	254
Hungary	171
Italy	517
Netherlands	209
Norway	468
Poland	247
Russia	269
Spain	274
Turkey	280
UK	268
Most common diagnoses	
Psoriasis	484
Eczema	239
Acne	185

	No. of patients	Average age (years, range)
All subjects	3542	46.29 (18–95)
Sex		
Male (*n*)	1558	47.76 (18–92)
Female (*n*)	1984	45.14 (18–95)
Average DLQI score^a^		6.69

EQ-5D domain (no. of patients)	No problems	Some problems	Extreme problems
Mobility	2692	839	11
Self-care	3162	372	8
Usual activities	2615	874	53
Pain or discomfort	1604	1739	199
Anxiety or depressed	1954	1431	157

^a^DLQI total score range is 0–30, 0 indicating no impairment and 30 indicating maximum impairment of quality of life

As no official European time trade-off (TTO) values exist for EQ-5D health states, we applied the UK TTO values throughout the validation process.

### Conceptual correlations

We assessed the strength of the conceptual correlations between the DLQI and EQ-5D and found that several key themes were significantly associated (i.e., *p* < 0.05). The key concepts that apply to each DLQI item are shown in Table 2.

**Table 2:** Key concepts that apply to each DLQI item [1]

Section	Items
Symptoms and feelings	1, 2
Daily activities	3, 4
Leisure	5, 6
Work and school	7
Personal relationships	8, 9
Treatment	10

For the ‘Mobility’ EQ-5D domain, DLQI items 3, 7, and 10 were most strongly correlated which cover the concepts of ‘daily activities,’ ‘work and school,’ and ‘treatment.’ The ‘Pain’ domain was strongly correlated with almost all key concepts of the DLQI including items 1, 3, 6, 8, 9, and 10. It correlated most with Item 1 of the DLQI, in particular, which asks about pain and soreness of the patient’s skin condition. The ‘Self-care’ domain correlated most strongly with item 10 (treatment), as well as items 1, 3, and 7. ‘Usual activities’ correlated strongly with item 3 (daily activities) as expected, as well as items 1, 5, 6, 7, and 10. Finally, the ‘Anxiety’ domain was most strongly correlated to item 2, which enquires about ‘embarrassment’ and whether patients feel ‘self-conscious’ due to their skin condition, as well as items 4, 5, 7, 9, 10. Overall, all ten DLQI items correlated strongly with the EQ-5D domains, re-emphasizing the strong conceptual correlation between the two questionnaires.

### Ordinal regression modeling algorithm

Ordinal models produce a set of probabilities for each possible outcome category, as given by the equations:

$$P\left( {Y = 1} \right) = \frac{1}{{1 + {\text{e}}^{{( - a_{1} + b_{1} x_{1} + b_{2} x_{2} + \cdots + b_{m} x_{m} )}} }}$$

$$P\left( {Y = 2} \right) = \frac{1}{{1 + {\text{e}}^{{( - a_{2} + b_{1} x_{1} + b_{2} x_{2} + \cdots + b_{m} x_{m} )}} }} - P(Y = 1)$$

$$P\left( {Y = 3} \right) = 1 - P\left( {Y = 2} \right) - P(Y = 1)$$

‘Y’ represents the outcome of any given EQ-5D domain (“mobility,” “self-care,” “usual activities,” “pain/discomfort,” or “anxiety/depression”). The outcome categories *Y* = 1, 2, and 3 represent the three possible responses for a given EQ-5D domain, i.e., “no problems,” “some problems,” or “extreme problems,” respectively. Sex was coded as 0 = male and 1 = female. The *x*-variables are indicator variables derived from DLQI scores, age, fitted as a linear term, and sex, while the *b*’s are the regression coefficients. The *b*’s are essentially ‘weights’ attached to each indicator of each DLQI item score, age, and sex and they are used to calculate estimated probabilities of each EQ-5D item response. The model is based on the assumption that for each EQ-5D dimension there is an underlying continuous ‘latent’ variable, for example, measuring mobility. The value of the linear combination $b{}_{1}x_{1} + b_{2} x_{2} + \cdots + b_{m} x_{m}$ provides a predicted score, *Z*, on this continuum. If we assume that these scores *Z* follow a logistic distribution, then the OLR model follows from assuming that if *Z* < *a*_1_, the subjects would record an outcome *Y* = 1, if *a*_1_ < *Z* < *a*_2_, they would record an outcome of *Y* = 2, and if *Z* > *a*_2_ they would record an outcome *Y* = 3.

Using all data, a series of ordinal logistic regressions were fitted for each of the five EQ-5D dimensions against the ten individual items of the DLQI, as well as age and sex using SPSS version 22. All ten DLQI items were included for each domain model in order to capture all the correlations induced by each DLQI item. Regressions were run with age and sex alone, DLQI items alone, as well as age and sex combined with DLQI items (Table 3) in order to evaluate the contribution of age and sex, and collectively the ten DLQI items. Model comparisons were undertaken by comparing twice the absolute difference in the maximized log-likelihoods with the Chi-square distribution with degrees of freedom equal to the difference in the number of model terms being evaluated. Note that age and sex were chosen as additional variables as these data are invariably recorded and therefore accessible and have been shown to significantly impact on QoL [18].

**Table 3:** The significance of the DLQI items and age and sex compared to the model containing age, sex, and the DLQI items for each EQ-5D domain

EQ-5D domain	Covariates: age/sex			Covariates: DLQI			Covariates: age/sex/DLQI
	−2 log likelihood	Chi-square comparing to full model	Degrees of freedom (*df*)	−2 log likelihood	Chi-square comparing to full model	Degrees of freedom (*df*)	−2 log likelihood
Mobility	507.4	171.9	2	1311	107.0	10	1566
Self-care	430.8	18.7	2	862.5	172.9	10	988.7
Usual activities	610.2	36.6	2	1388.1	269.2	10	1754.1
Pain	783.8	37.5	2	1738	424.9	10	2373.3
Anxiety/depression	772.6	19	2	1787.9	284.4	10	2451.7

### Model validation

Split-half cross-validation was employed [19] whereby the dataset was randomly split five times into separate estimation and validation sets using the random number generator in SPSS version 22. The estimation set was used to derive the mapping models, whilst the out-of-sample validation set was utilized for validating the fitted models. This process was repeated with each of the five estimation/validation sets after which the sets were reversed, resulting in a total of 10 complete models.

Bootstrapping has been suggested as an alternative approach to model validation [19] although that technique was evaluated in a somewhat simpler setting than the one considered here, namely with a single binary outcome variable and a single logistic model rather than with five ordinal outcomes and a separate logistic model in each case. As these authors note, however, bootstrapping is likely to offer relevant advantages in datasets with small sample sizes. The issue of small sample sizes and bootstrapping is discussed further in relation to model validation [20] when predictor selection techniques have been employed. In our case, the sample size is sufficiently large and there is no predictor selection, supporting the use of split-half cross-validation.

The model was tested on each validation dataset to produce three predicted probabilities per subject per EQ-5D domain (*Y* = 1, 2, or 3). Using these predicted probabilities, a Monte Carlo simulation was run for each subject resulting in predicted domain responses and consequently health utility estimates. This was repeated five times for each random split to ensure the model output was stable. The five estimation and validation sets were then switched and the process was repeated (split-half cross-validation), resulting in a total of ten models. The average predicted health utility estimate for each validation set was then compared with the observed health utility estimate of the same set.

The proportional odds assumption was assessed using the test for parallelism within SPSS. For each domain, except mobility, this test gave a non-significant result supporting the assumption for proportional odds. For mobility, the *p* value of 0.01 did indicate some departure from this assumption but this can be explained by the small number of subjects (*n* = 11) in the dataset who have a mobility outcome category of 3. As a consequence, the sub-model that compares categories 1 and 2 combined with category 3 is unstable and the results for the test for parallelism unreliable.

### Currie and Conway method: linear regression

The methodology reported above for model derivation, split-half cross-validation, and Monte Carlo simulation was repeated to test the linear regression algorithm utilized by Currie and Conway [9]. This method uses the total DLQI scores and correlates it directly with the final health utility estimates resulting in a linear regression formula in the format: Utility = *a* − (*b* × DLQI total score).

The average difference between observed health utility estimates and predicted health utility estimates was calculated for both OLR and linear regression methods, as well as mean square error (MSE) and mean absolute error (MAE).

## Results

### Model validation

#### OLR method

For each of the five EQ-5D domains, an ordinal model was derived and used to predict the probability of each EQ-5D response for each subject in each validation set, and subsequently the health utility estimates, using Monte Carlo simulation. The model was shown to be highly predictive, and repeated data splits demonstrated a stable model. In each case, the predicted mean health utility estimate was a slight underestimate of the observed mean health utility estimate and across the ten validation sets, the difference between predicted mean health utility estimates and observed mean health utility estimates ranged from −0.0024 to −0.0239, with an mean overall difference of −0.0120. This 1.59% underestimate represents a clinically unimportant effect [21]. The MSE across all ten splits ranged from 0.0728 to 0.0818 with an average MSE of 0.0766. The MAE across all ten splits ranged from 0.1873 to 0.2009 with an average MAE of 0.1934.

**Fig. 1:**
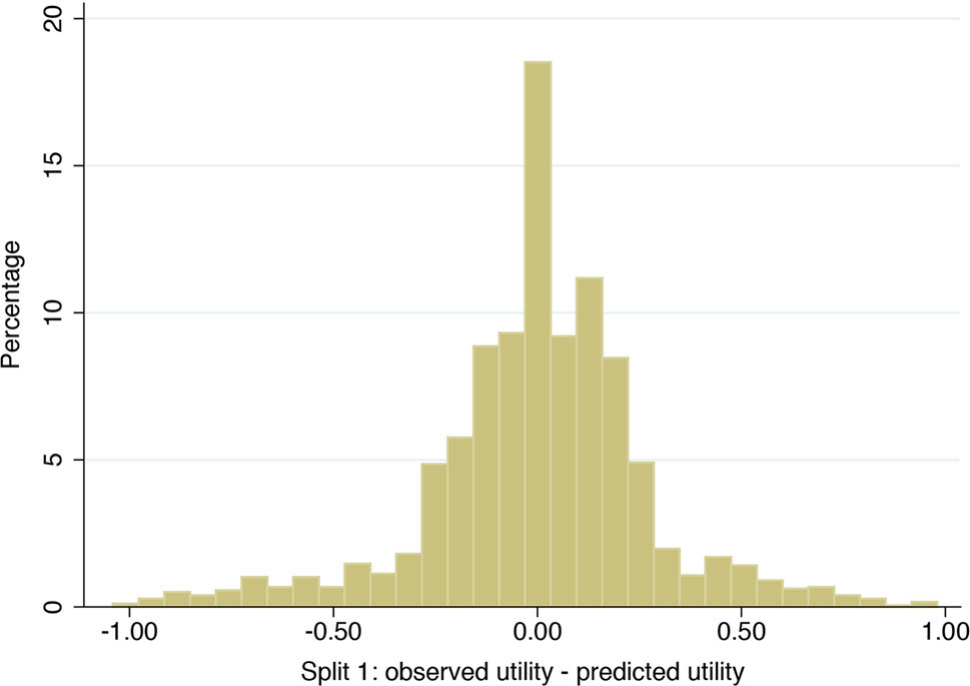
Histogram displaying the difference between predicted and observed health utility estimates for a typical split sample

The predictive ability of the model at an individual subject level was also examined using histograms to display the difference between predicted health utility estimates and the observed health utility estimates for each simulation at the individual subject level. The results from a typical split sample are displayed in Fig. 1. The plot depicts a centrality around ‘0’ which indicates the strong predictive collective capability of the OLR models. On average, 37% of the individual health utility estimates were predicted to lie within 0.1 of the observed values, while 62% were predicted to lie within 0.2 and 81% within 0.3 over all 10 validation exercises.

To further evaluate its reliability, the OLR mapping method was also applied to different subsets of the study population. A model was derived from psoriasis-only patients (*n* = 484) and tested on patients with all other skin conditions (*n* = 3058). The average difference between the observed and predicted health utility estimates was 0.05 (MSE 0.0844, MAE 0.2037). Thirty-six percent of the individual health utility estimates were predicted to lie within 0.1 of the observed values, while 61% were predicted to lie within 0.2 and 78% within 0.3.

Similarly, the model performance was tested on different geographical groups of patients. As a test exercise, a model derived from patients in Italy (*n* = 517) was tested on patients from Norway (*n* = 468). The average health utility estimate difference for the Norway patients was 0.06 (MSE 0.09. MAE 0.21). Thirty-six percent of the individual health utility estimates were predicted to lie within 0.1 of the observed values, while 59% were predicted to lie within 0.2 and 78% within 0.3.

Despite the small sample sizes for the model building exercise in these two cases, these evaluations support the reliability and robustness of the modeling framework.

Details of the final-fitted OLR models using data from the 3542 subjects are given in Table 4.

**Table 4:** Final model estimates (standard errors) for each EQ-5D domain

	Mobility	Self-care	Usual activities	Pain/discomfort	Anxiety/depression
Threshold a_1_	4.500 (0.190)	4.854 (0.251)	3.574 (0.171)	2.204 (0.133)	1.469 (0.128)
Threshold a_2_	9.506 (0.368)	9.074 (0.438)	7.231 (0.237)	6.052 (0.178)	4.775 (0.162)
Age	0.051 (0.003)	0.033 (0.004)	0.027 (0.003)	0.025 (0.002)	0.003 (0.002)
Sex^a^	0.046 (0.089)	−0.213 (0.120)	0.133 (0.087)	0.177 (0.073)	0.465 (0.073)
DLQI 1	0.087 (0.055)	0.176 (0.074)	0.270 (0.052)	0.685 (0.047)	0.035 (0.044)
DLQI 2	0.013 (0.061)	0.052 (0.079)	−0.114 (0.059)	0.014 (0.049)	0.378 (0.048)
DLQI 3	0.209 (0.068)	0.278 (0.085)	0.351 (0.063)	0.199 (0.060)	0.107 (0.057)
DLQI 4	0.071 (0.058)	0.053 (0.072)	0.051 (0.055)	0.097 (0.050)	−0.099 (0.048)
DLQI 5	0.113 (0.075)	0.064 (0.095)	0.209 (0.070)	−0.122 (0.064)	0.205 (0.062)
DLQI 6	0.116 (0.060)	0.014 (0.071)	0.215 (0.055)	0.310 (0.054)	−0.075 (0.052)
DLQI 7	0.251 (0.053)	0.236 (0.063)	0.283 (0.049)	−0.048 (0.046)	0.186 (0.044)
DLQI 8	−0.008 (0.076)	−0.013 (0.091)	−0.081 (0.071)	0.163 (0.066)	0.121 (0.064)
DLQI 9	−0.094 (0.065)	0.002 (0.075)	0.068 (0.060)	0.132 (0.057)	0.194 (0.054)
DLQI 10	0.233 (0.061)	0.478 (0.071)	0.210 (0.057)	0.245 (0.054)	0.155 (0.052)

The 10 DLQI questions are represented in order by DLQI 1, DLQI 2, etc

^a^Sex was coded male = 0, female = 1

### Currie and Conway method

For the Currie and Conway linear regression model, the average difference between the observed and predicted estimates was −0.0007. The MSE across all ten splits ranged from 0.0438 to 0.05 with an average MSE of 0.0469. The mean absolute error (MAE) across all ten splits ranged from 0.1527 to 0.1616 with an average MAE of 0.1566. On average, 38% of the individual health utility estimates were predicted to lie within 0.1 of the observed estimates, while 78% were predicted to lie within 0.2 and 89% within 0.3 over all 10 validation exercises.

## Discussion

There is increasing interest in correlating and mapping DLQI scores into generic measures, such as the EQ-5D, for cost-analysis and to provide more accurate disease-specific data which generic measures are unable to capture. Schmitt et al. [22] correlated the Work Limitations Questionnaire with the DLQI (*r* = 0.47, *p* < 0.0001) to derive a model to calculate work productivity in psoriasis. Moller et al. [23] state that ‘disutility among psoriasis patients are within the ranges of other chronic diseases.’ There is, therefore, a need to accurately represent and compare data from dermatology with health utility estimates from other conditions. Furthermore, there are several inherent disadvantages with generic measures [24] such as the EQ-5D or Short-Form 36 (SF-36), e.g., they contain irrelevant questions for patients with severe inflammatory skin conditions, resulting in the inability to perform imputation due to systematically missing responses in the questionnaires. Patients may also develop ‘questionnaire fatigue’ from repeated completions. Focusing on one specialty- or disease-specific questionnaire, from which health utility estimates may be predicted, provides a perception of relevance encouraging thorough careful completion by patients whilst also reducing study time and costs for researchers. Using OLR, this study has succeeded in mapping DLQI scores to EQ-5D data, from which health utility estimates were calculated. The model reliably predicts EQ-5D scores, in particular at a group level, demonstrated through a split-half cross-validation process resulting in very close health utility estimate predictions. The model is shown also to provide close prediction of health utility estimates at an individual subject level.

There are strong conceptual associations between the DLQI and EQ-5D items. Mapping is more likely to be successful where conceptual overlap between two measures exists [25]. This is so for the DLQI and EQ-5D; many studies have reported a strong association [26–31], which is reaffirmed by this study. Although overall predictions were strongly correlated to the observed scores at a group level, the individual predicting power of the model requires further testing.

The linear regression model utilized by Currie and Conway [9] provided better predictive accuracy when fitted on this study’s dataset (average difference between predicted and observed health utility estimates = 0.00065, compared to OLR = 0.0120). This was also reflected in the respective MAE (linear regression = 0.16, OLR = 0.19) and MSE (linear regression = 0.05, OLR = 0.08) values. It is therefore plausible that this mapping method performs better when fitted on a larger and dermatologically diverse dataset, compared to its previous validation study which was limited to a small sample size and to psoriasis patients in the UK [9]. However, there is one structural advantage in the use of the ordinal model over the linear model [9]. Since the DLQI total score always takes a positive value, the maximum utility value derived from the linear regression equation has an upper bound of ‘a.’ In a typical application, the value of the constant ‘a’ will approach 1 but will never be equal to 1 and a predicted health utility estimate of ‘1’ (‘perfect health’) cannot be obtained. In the OLR model and the associated Monte Carlo simulation such an outcome can be achieved. Both models’ estimates are derived from a European dataset of over 3500 patients with various dermatological conditions, and the predicted responses may be used to calculate country-specific health utility estimates [32]. This was not possible using the previous linear model [9], derived from a UK dataset, because of differing health utility estimate tariffs between countries [33, 34]. Thus the proposed ordinal model, as well as the revised linear regression model, may be used as mapping tools in other European countries.

There are some limitations that apply to both models. The observed scores for the DLQI and the EQ-5D were sometimes inconsistent within the same subject, e.g., one subject answered 1 on every EQ-5D domain (‘perfect health’) but 29 on the DLQI (very poor health). This could be due to poor understanding of the items, the reliability or validity of the instruments, or due to random errors. Though these data were included to avoid bias, Van Hout et al. [35] argue that analysis should be restricted to logically consistent responses. Perhaps including more socio-demographic variables in the OLR model, other than age and sex, may improve its predictive performance, though this may result in only marginal improvements that would not outweigh the complexity of running the model [15]. The UK TTO values were used in the derivation of both models; it is worth considering that these health states were elicited in 1993 and therefore may not be up to date with current health valuations. Furthermore, no official European TTO values exist for EQ-5D health states and therefore we applied the UK TTO values throughout the validation process. Further sensitivity analysis may be conducted using preference value-sets from different countries. However, these were not accessible for this study, but would be a useful consideration for future studies. Though there may be cultural variation influencing HRQoL and utility responses, we have not been able to test this specific question in detail. However, when the OLR model was created using only Italian patients and tested on a Norway population, it performed almost as well as the model derived from the complete dataset. Our experience suggests that within the European context there is some uniformity of attitudes, cultural norms, and responses, as the DLQI has undergone over one hundred validated translations, with a significant number in European countries [2]. We believe the methodology remains intact and consistent, regardless of the TTO values utilized.

Though bootstrapping may indeed be the best approach for testing such models, this would require some additional theoretical considerations to extend existing methodology for the binary logistic model to the ordinal setting. We were able to bypass this approach by using ‘split-half cross-validation,’ which is a valid technique for large sample sizes [19]. Nevertheless, this study presents the opportunity for further statistical research.

There may be concerns regarding the use of these models in different diseases and whether single disease models would provide more accurate utility data. This study includes a wide range of the most common different skin diseases from a wide range of different European countries, giving the models additional strength in terms of universality. However, we successfully derived a model from psoriasis-only patients and tested this on patients with all other conditions, with the predicted results reassuringly similar to the original OLR model validation exercise. Two limitations of this exercise were the sample size of psoriasis patients, which was relatively small (*n* = 484) and that none of the patients had answered ‘extreme’ for the self-care domain of the EQ-5D. Given the overall sample size from which the OLR model was created, our view is therefore that the model may be implemented successfully across different conditions, limiting the need for condition-specific modeling, which may be practically difficult to create.

Though we initially hypothesized that OLR will improve upon previous attempts at predicting health utility estimates, we have identified that both of the existing templates may be used as a road map across other medical disciplines in instances where similar needs exist. Both methodologies will therefore be useful for researchers interested in deriving generic HRQoL data, including descriptive information, from disease-specific populations without having to implement numerous questionnaires. Though OLR has previously been used for converting measures [15], as far as we are aware this is first time it has been used to convert a specialty-specific instrument into a generic measure. A step-by-step guide is provided to implement the OLR model (Supplementary material) in the particular setting of mapping the DLQI scores to EQ-5D health utility estimates. An excel spreadsheet is also available upon request with pre-programmed formulae to enable EQ-5D domain probability calculations for a cohort of patients, from which health utility estimates may be predicted using Monte Carlo simulation. The DLQI is the most commonly reported outcome measure in dermatology [2, 36], and therefore there are many datasets from which generic EQ-5D and health utility data can now be predicted, using either OLR or linear regression.

### Electronic supplementary material

Below is the link to the electronic supplementary material.

Supplementary material 1 (DOCX 21 kb)

## Appendix

The Dermatology Life Quality Index [1, 16]*

**Table:** The scoring of each question is as follows

Response	Score
Very much	Scored 3
A lot	Scored 2
A little	Scored 1
Not at all	Scored 0
Not relevant	Scored 0
Question unanswered	Scored 0
Question 7: “prevented work or studying”	Scored 3
