# Supplementary material for: Mapping of the DLQI scores to EQ-5D utility values using ordinal logistic regression
Source: Qual Life Res. 2017 Jun 10;26(11):3025–34. doi: 10.1007/s11136-017-1607-4 (PMC5655589; doi:10.1007/s11136-017-1607-4)
Supplement: Supplementary file 1 — Supplementary material 1 (DOCX 21 kb) [file 11136_2017_1607_MOESM1_ESM.docx]

**Supplementary Material – Guide to using the fitted Ordinal Logistic Regression model to predict utility values from predicted EQ-5D domain scores derived from DLQI item scores**

The purpose of this appendix is to provide a practical guide to other researchers to use the model described in this paper. It is assumed that the researcher has a data set of DLQI values (each with 10 individual question scores), as well as age and sex data, from a population of subjects, and that the researcher wishes to create predicted utility values from predicted EQ-5D individual domain scores for that population.

The coefficients that are used in the fitted model are given in Table 3. Consider initially the EQ-5D domain ‘Mobility’ (column 2 of Table 3 gives the coefficients).

**Step 1**

All ten DLQI items, the patient’s age (years) and the patient’s sex (male = 0, female = 1) are contained in each of the 5 models. For mobility, for example, for each subject we first calculate the latent variable (from Table 3, column 2):

$$b_{1}x_{1}+b_{2}x_{2}+\ldots+b_{m}x_{m}$$

(where the *b’s* are the coefficients and the *x’s* are the indicator variables relating to the DLQI items, age and sex)

Suppose for this subject (male, aged 37) the observed DLQI item scores are respectively 1 (item 1), 1 (item 2), 0 (item 3), 1 (item 4), 2 (item 5), 1 (item 6), 3 (item 7), 0 (item 8), 1 (item 9) and 2 (item 10). Taking values from Table 3, the latent variable value is then:

(37 x 0.051) + (0 x 0.046) + (1 x 0.087) + (1 x 0.013) + (0 x 0.209) + (1 x 0.071) + (2 x 0.113) + (1 x 0.116) + (3 x 0.251) + (0 x -0.008) + (1 x -0.094) + (2 x 0.233) = 3.525

**Step 2**

The probability that this subject falls into category 1 (denoted $P(Y=1))$of the EQ-5D domain for ‘Mobility’ is:

$$P\left( Y=1 \right)=\frac{1}{1+e^{(-\left( 4.500 \right)+3.525)}}=0.726$$

Note that 4.500 here is the $a_{1}$threshold for mobility in Table 3. The probability that this subject falls into either category 1 or 2 of the EQ-5D domain for ‘Mobility’ is then:

$$P\left( Y=1 \right)+P\left( Y=2 \right)=\frac{1}{1+e^{\left( -\left( 9.506 \right)+3.525 \right)}}=0.997$$

Here 9.506 is the $a_{2}$ threshold for mobility and it follows by subtraction of $P\left( Y=1 \right)$ from $P\left( Y=1 \right)+P(Y=2)$ that $P\left( Y=2 \right)=0.271$. Finally, since the probability values for $Y=1, Y=2 and Y=3,$ sum to 1, $P\left( Y=3 \right)=1-0.997=0.003$

**Step 3**

Repeat this calculation giving $P\left( Y=1 \right), P\left( Y=2 \right) and P(Y=3)$ for each subject and then use Monte Carlo simulation to assign an outcome for the ‘Mobility’ domain based on the calculated probabilities.

**Step 4**

Repeat for each of the five EQ-5D domains.

**Step 5**

With predicted scores for all five domains now obtained for all patients, cross-check with country-specific TTO value sets (available upon request from <http://www.euroqol.org>) to derive utility values. For larger datasets, SPSS syntaxes are also available from <http://www.euroqol.org>. Utility values for the entire dataset may then be averaged to provide an overall reflection of the dataset’s generic health state.

An Excel spreadsheet is available from the corresponding author (Dr Faraz Ali, alifm@cardiff.ac.uk) to be used to make the calculations in **Steps 1-4**.
